# Faecal short-chain fatty acids - a diagnostic biomarker for irritable bowel syndrome?

**DOI:** 10.1186/s12876-016-0446-z

**Published:** 2016-04-27

**Authors:** Per G. Farup, Knut Rudi, Knut Hestad

**Affiliations:** Department of Research, Innlandet Hospital Trust, N-2381 Brumunddal, Norway; Unit for Applied Clinical Research, Department of Cancer Research and Molecular Medicine, Faculty of Medicine, Norwegian University of Science and Technology, N-7491 Trondheim, Norway; Department of Chemistry, Biotechnology and Food Science, Norwegian University of Life Sciences, P.O. Box 5003, , N-1432 Ås, Norway; Department of Psychology, Faculty of Social Sciences and Technology Management, Norwegian University of Science and Technology, N-7491 Trondheim, Norway; Department of Public Health, Hedmark University College, N-2418 Elverum, Norway

## Abstract

**Background:**

The diagnosis of irritable bowel syndrome (IBS) relies on symptom-based criteria. A valid and reliable biomarker that could confirm the diagnosis is desirable. This study evaluated the properties of faecal short-chain fatty acids (SCFA) as diagnostic biomarkers for IBS.

**Methods:**

Twenty-five subjects with IBS and 25 controls were included in this explanatory case–control study. Stool samples were analysed for SCFA (acetic acid, propionic acid, butyric acid, isobutyric acid, valeric acid, and isovaleric acid) with gas chromatography and reported as mmol/l and molar%. In the search for the best way to distinguish between subjects with and without IBS, the total amount and the amount of each of the SCFA were measured, and the proportions and differences between the SCFA were calculated.

**Results:**

In the IBS and control group, the mean age was 46.2 (SD 12.9) and 49.2 (SD 14.6), and the number of females was 13/25 (52 %) and 15/25 (60 %) respectively. The difference between propionic and butyric acid (mmol/l) had the best diagnostic properties, the area under the Receiver Operating Characteristic curve was 0.89 (95 % CI: 0.80–0.98) (*p* < 0.001). With a cut-off value > 0.015 mmol/l indicating IBS, the sensitivity, specificity, positive and negative likelihood ratio, and diagnostic odds ratio were 92 %, 72 %, 3.29, 0.11 and 29.6 respectively. Similar diagnostic properties were shown for all the IBS subgroups.

**Conclusions:**

The study indicated that faecal SCFA could be a non-invasive, valid and reliable biomarker for the differentiation of healthy subjects from subjects with IBS.

## Background

Irritable bowel syndrome (IBS) is a common functional gastrointestinal disorder [1, 2]. The diagnosis is based on internationally accepted symptom-based criteria (the Rome criteria) after exclusion of organic diseases [3]. Since multiple disorders have similar symptoms, a valid and reliable diagnostic biomarker for IBS has been highly demanded. Physicians often avoid setting a positive diagnosis of IBS without extensive investigations. Clarke et al.’s statement in 2009 has the same actuality today: “The successful identification of biomarkers is critical to progressing our understanding of IBS and addressing the unmet therapeutic needs of this debilitating condition” [4].

Extensive research has been carried out to find valid and reliable biomarkers for IBS. Several biomarkers separate IBS from organic diseases (e.g. Inflammatory Bowel Disease) and healthy volunteers, but so far none has been judged as satisfactory for the use in daily practice [5, 6].

The association between IBS and the gut microbiota has been demonstrated in several studies and efforts have been made to characterise the abnormal microbiota in patients with IBS [7, 8]. So far, the results have been inconsistent. For the human health and disease, the function of the microbiota might, therefore, be as important as the phylotype. It has been argued that “the phylotype provides the environmentally selected interface for the functions”[9]. The function could be measured as chemicals and metabolites in the faeces. The microbiota metabolises non-digestible food constituents into short-chain fatty acids (SCFA) that have extensive immunological and regulatory functions and appear to be the link in the host-microbe interactions [10–13]. The relation between functional characteristics, such as SCFA and functional bowel disorders has not been extensively investigated. The immunological activation often seen in subjects with IBS could be associated with the changes in SCFA [11, 14–17]. In one study, volatile organic metabolites showed promising diagnostic properties for IBS [16].

The aims of this case–control study were to compare faecal SCFA in subjects with and without IBS and search for biomarkers that could aid in differentiating patients with IBS from healthy subjects.

## Methods

### Design

This study was an add-on to a previous study comparing patients with depression and unspecified neurological symptoms [18, 19]. Fifty patients, 25 with IBS and 25 without, were arbitrarily selected for this case–control study.

### Participants

In the previous study, patients above 17 years of age with the diagnosis of idiopathic depression (according to ICD-10; F 32–34 spectre) were included in the “depression group”. Patients admitted to an inpatient neurological clinic with unexplained neurological symptoms were included in the “neurological group”. In both groups, organic diseases were excluded after comprehensive clinical, laboratory, and supplementary investigations according to the doctors’ discretion. In the neurological group, the examinations included a thorough neurological examination, analysis of the cerebrospinal fluid and either a cerebral CT or MRI scan. Subjects with excessive alcohol intake were excluded. Inclusion and exclusion criteria, design and methods have been described in detail in previous papers [18, 19].

### Variables

*Demographics:* Gender; Age (years); education (number of years in school).

*Abdominal complaints:* IBS (yes/no) according to the Rome III criteria was assessed with a validated Norwegian translation of the Rome-questionnaire [3].

*Depression:* Beck Depression Inventory v. II (BDI II) (minimal, mild, moderate and severe depression; scores 0–13, 14–19, 20–28 and 29–63 respectively) [20]. Montgomery-Åsberg Depression Scale (MADRS): (symptom absent, mild, moderate and severe depression; scores 0–6, 7–19. 20–34 and 35–60 respectively) [21].

*Faecal samples:* Stool samples were analysed for short-chain fatty acids (acetic acid, propionic acid, butyric acid, isobutyric acid, valeric acid, and isovaleric acid) with gas chromatography as described by Szczesniak et al. [22]. The amounts of SCFA have been reported as mmol/l and molar%. In the search for the best way to distinguish subjects with and without IBS, the amount of SCFA and the proportion and differences between the SCFA were used.

### Statistics

The exact chi-square test and Student *t*-test were used for the comparisons between the groups, and non-parametric statistics was used in addition for variables without normal distribution. Linear multiple regression was used to study independent predictors. The diagnostic properties were assessed with Receiver Operating Characteristics (ROC) curves, sensitivity, specificity, positive- and negative Likelihood Ratio (LR) and Diagnostic Odds Ratio (DOR). *P*-values below 0.05 were judged as statistically significant.

### Ethics

The study was approved by the Norwegian Regional Committees for Medical and Health Research Ethics, PB 1130, Blindern, 0318 Oslo, Norway (reference number 2009/2196a) and performed in accordance with the Declaration of Helsinki. Written informed consent was given by all participants before inclusion.

## Results

### Participants

Twenty-five Caucasian subjects with IBS and 25 without were included in the study. Table 1 gives the characteristics of the subjects. No patients were excluded from the study due to an organic disorder that could explain the IBS-like symptoms, and none used antibiotics. The number of subjects with constipation-predominant IBS (IBS-C), diarrhoea-predominant IBS (IBS-D), mixed IBS (IBS-M) and undefined IBS (IBS-U) was 8, 9, 7 and 1 respectively. Except for higher scores for depression in the IBS group than in the control group, the groups were well balanced. The imbalance in the degree of depression occurred by chance and was due to a higher proportion of subjects from the “depression group” in the IBS group (19/25; 76 %) than in the control group (14/25; 56 %).

**Table 1:** The participants’ characteristics

Characteristics	IBS (no 25)	Control (no 25)	Statistics (*p*-values)
Gender (female/male)	13 (52 %)/12 (48 %)	15 (60 %)/10 (40 %)	ns (*p* = 0.78)
Age (years)	46.2 (12.9)	49.2 (14.6)	ns (*p* = 0.45)
Group (depression/neuro)	19 (76 %)/6 (24 %)	14 (56 %)/11 (44 %)	ns (*p* = 0.23)
Education (years)	13.0 (2.8)	13.1 (2.5)	ns (*p* = 0.83)
BDI II (score)	26.7 (15.6)	14.2 (9.5)	*p* = 0.001
MADRS (score)	24.8 (11.4)	15.5 (9.4)	*p* = 0.003

The results are given as the number with proportion (%) in brackets and mean with SD in brackets

*BDI II* Beck Depression Inventory v. II

*MADRS* Montgomery-Åsberg Depression Scale

### SCFA

Table 2 gives the results of the SCFA with comparisons between subjects with and without IBS. Butyric acid (molar%) was statistically significantly higher in the control group, and there was a non-significant trend toward a higher concentration of propionic acid (mmol/l and molar%) in the IBS group. Therefore, the propionic/butyric ratio and the differences between propionic acid and butyric acid (Prop-But) were calculated. They showed highly statistically significant differences between the groups. The best one for the discrimination between subjects with and without IBS turned out to be Prop-But (mmol/l). This variable also showed statistically significant differences between the three subgroups of IBS (IBS-C, IBS-D and IBS-M) and the control group (Table 3).

**Table 2:** Faecal short-chain fatty acids in subjects with and without IBS with comparisons between the groups

Short-chain fatty acids (SCFA)	IBS (no 25)	Control (no 25)	Statistics (*p*-values)
The sum of SCFA (mmol/l)	10.54 (9.14)	8.36 (7.57)	ns (*p* = 0.36)
Acetic acid (mmol/l)	6.42 (6.29)	4.81 (4.98)	ns (*p* = 0.32)
Propionic acid (mmol/l)	1.93 (1.43)	1.34 (0.98)	ns (*p* = 0.10)
Butyric acid (mmol/l)	1.56 (1.25)	1.67 (1.56)	ns (*p* = 0.80)
Iso-Butyric acid (mmol/l)	0.17 (0.10)	0.15 (0.12)	ns (*p* = 0.52)
Valeric acid (mmol/l)	0.19 (0.13)	0.17 (0.13)	ns (*p* = 0.42)
Iso-Valeric acid (mmol/l)	0.25 (0.13)	0.22 (0.14)	ns (*p* = 0.34)
Acetic acid (molar%)	56.26 (7.56)	55.48 (8.23)	ns (*p* = 0.73)
Propionic acid (molar%)	20.20 (4.95)	17.85 (3.94)	ns (*p* = 0.07)
Butyric acid (molar%)	15.58 (2.75)	19.00 (4.55)	*p* = 0.003
Iso-Butyric acid (molar%)	2.21 (1.07)	2.10 (1.42)	ns (*p* = 0.75)
Valeric acid (molar%)	2.25 (0.74)	2.08 (1.00)	ns (*p* = 0.49)
Iso-Valeric acid (molar%)	3.50 (2.04)	3.50 (1.98)	ns (*p* = 0.99)
Propionic – Butyric acid (mmol/l)	0.36 (0.34)	- 0.32 (0.69)	*p* < 0.001
Propionic – Butyric acid (molar%)	4.61 (5.22)	- 1.15 (5.46)	*p* < 0.001
Propionic/Butyric mmol/l ratio (%)	132 % (37 %)	98 % (27 %)	*p* < 0.001

The results are given as mean values with SD in brackets. The Student *T*-test was used for the comparisons between the groups. Several of the variables were not normally distributed. Analyses with non-parametric statistics (Mann–Whitney *U*-test) showed principally identical results (data not shown)

**Table 3:** The “Propionic minus Butyric acid (mmol/l)” values in the subgroups of subjects with IBS and comparisons with the control group

IBS subtype	Propionic minus Butyric acid		Statistics *p*-value
	IBS	Control	
IBS-C (no 8)	0.21 (0.24)	- 0.32 (0.69)	*p* = 0.04
IBS-D (no 9)	0.51 (0.37)	- 0.32 (0.69)	*p* = 0.002
IBS-M (no 7)	0.37 (0.37)	- 0.32 (0.69)	*p* = 0.016
IBS-U (no 1)	0.24 (−−-)	- 0.32 (0.69)	ns (*p* = 0.43)

*Abbreviations*: IBS-C: Constipation-predominant IBS; IBS-D: Diarrhoea-predominant IBS; IBS-M: Mixed IBS; IBS-U: Undefined IBS. No: Number of subjects

The results are given as mean values with SD in brackets. The Student *T*-test was used for the comparisons between the groups. Several of the variables were not normally distributed. Analyses with non-parametric statistics (Mann–Whitney *U*-test) showed principally identical results (data not shown)

The imbalance between the groups concerning depression made it necessary to control for confounding. A linear regression analysis with Prop-But as the dependent variable, and group (IBS/control) and BDI II as covariates was performed. IBS, but not BDI II was an independent predictor of Prop-But; standardised beta and *p*-values were 0.55, *p* < 0.001; and - 0.01, *p* = 0.92; respectively.

### Diagnostic properties of SCFA

**Fig. 1:**
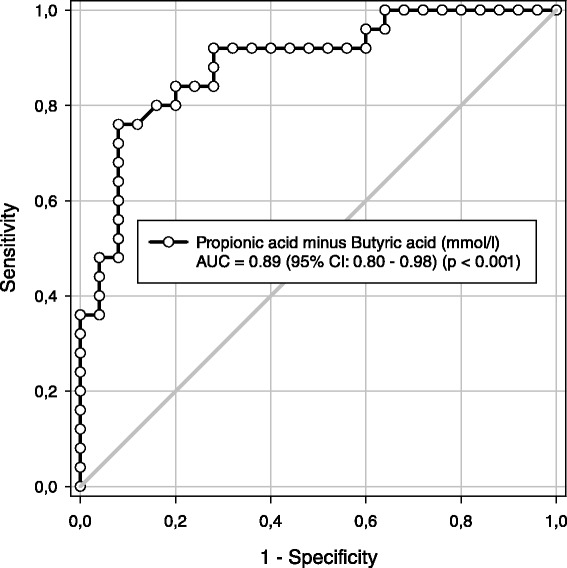
The diagnostic properties of Propionic – Butyric acid (mmol/l) presented as the Receiver Operating Characteristic curve

The differences between subjects with and without IBS were highly statistically significant for the “Propionic acid/Butyric acid ratio” and “Propionic acid minus Butyric acid (mmol/l and molar%)” (Prop-But) (Tables 2 and 3). Prop-But (mmol/l) showed the best diagnostic properties. Figure 1 shows the diagnostic property of Prop-But (mmol/l) presented as the Receiver Operating Characteristics (ROC) curve. The area under the curve (AUC) was 0.89 (95 % CI 0.80: 0.98), *p* < 0.001. A Prop-But value > 0.015 was judged as a well-suited cut-off for a positive test for IBS and was used in the calculation of the diagnostic properties. Table 4 gives the diagnostic properties of Prop-But (mmol/l) (AUC, sensitivity, specificity, positive- and negative likelihood ratio and diagnostic odds ratio) for all patients and for each of the IBS subgroups. A Prop-But value < - 0.13 excluded IBS (the sensitivity was 100 %), and a value > 0.46 confirmed the diagnosis of IBS (the specificity was 100 %).

**Table 4:** The diagnostic properties of “Propionic acid minus Butyric acid (mmol/l)” for the diagnosis of IBS in all subjects and the subgroups of IBS

IBS group	AUC (95 % CI)	Significance	Sens	Spec	Pos. LR	Neg. LR	DOR
IBS (all subjects)	0.89 (0.80:0.98)	*p* < 0.001	92 %	72 %	3.29	0.11	29.6
IBS-C (no 8)	0.83 (0.67:0.99)	*p* = 0.006	87.5 %	72 %	3.13	0.17	18.0
IBS-D (no 9)	0.92 (0.78:1.00)	*p* < 0.001	89 %	72 %	3.17	0.15	20.6
IBS-M (no 7)	0.91 (0.81:1.00)	*p* = 0.001	100 %	72 %	3.57	0.00	NA
IBS-U (no 1)	0.92 (0.81:1.00)	ns (*p* = 0.16)	100 %	72 %	3.57	0.00	NA

*Abbreviations*: IBS-C: Constipation-predominant IBS; IBS-D: Diarrhoea-predominant IBS; IBS-M: Mixed IBS; IBS-U: Undefined IBS; AUC: Area Under the Curve; CI: Confidence interval; Sens.: Sensitivity; Spec: Specificity; Pos. LH: Positive Likelihood Ratio; Neg. LR: Negative Likelihood Ratio; DOR: Diagnostic Odds Ratio

## Discussion

There is a pressing need for a good biomarker for IBS. The list of attempts to find a suitable biomarker is long and has increased the understanding of IBS, but not resulted in a tool for use in daily practice [5, 6]. It was recently stated that no tool has surpassed the symptom-based criteria [5]. This study indicated that faecal SCFA could be a valuable diagnostic biomarker for IBS. With the chosen cut-off value, a negative test nearly excluded IBS.

The evidence for an association between the gut microbiota and IBS is increasing, at least in a subpopulation of the patients [7, 8, 23]. The microbiota is presumed to induce pathophysiological reactions such as activation of the mucosal immune system, increased intestinal permeability, activation of sensory pathways, and modulation of the enteric motility. Several studies show differences, but not the same differences, between the microbiota in patients with IBS and healthy volunteers and between subgroups of patients with IBS [8]. The findings indicate an imbalance of the microbiota (dysbiosis) in subjects with IBS, but a precise description of the imbalance has so far not been successful.

Knowledge of the function of the microbiota might be as relevant as knowledge of the microbiota itself and might add diagnostic and mechanistic insight to the study of the composition of the microbiota [12, 13, 23]. SCFA are produced by the microbiota trough fermentation of ingestible polysaccharides and proteins, and have been described as the link between the microbes and the host [10, 11]. SCFA have anti-inflammatory effects by modulation of the immune cell chemotaxis, and the release of reactive oxygen species (ROS) and cytokines [10]. The effects are mediated mainly by inhibition of histone deacetylases (HDACs) and stimulation of G-protein-coupled receptors (GPCRs), particularly GPR43 [10, 12]. IBS might be an inflammatory disorder. Studies in subjects with IBS have shown an activation of the immune system and an imbalance in the cytokine pattern [14, 15]. The lack of association between the total amount of SCFA in subjects with and without IBS seen in this and other studies could be due to different and opposite effects of the SCFA [11, 23–26]. The exact pathophysiology of IBS is unknown, and the absolute and relative amount of each of the SCFA might be more important than the total amount of SCFA. Butyrate, which is a key promoter of colonic health and the main provider of energy for the colonocytes, inhibits Il-12 and increases Il-10 production, and has a beneficial effect in various disorders [11, 13, 27]. Propionate is a potent activator of GPR43 that is present in immune, nervous and endocrine cells along the entire gastrointestinal tract [10]. The effects of the SCFA vary significantly, and the variation in absolute and relative amount could explain the seemingly different associations between SCFA and diseases [11–13]. The unfavourable effect of propionic acid in subjects with IBS, and the reverse effects of propionic and butyric acid have been reported in some but not all studies [23–26]. The discrepancies between the studies could be caused by dietary variations such as variation in the intake of FODMAPs [17]. The findings in this study and other studies indicate that SCFA, particularly the relation between propionate and butyrate, could be a diagnostic biomarker of IBS.

In this study, SCFA showed very satisfactory diagnostic properties for the diagnosis of IBS. The best parameter was Prop-But (mmol/l) with AUC = 0.89, sensitivity 92 % and specificity 72 %. The results seem to be as good as other diagnostic tools, but comparisons are difficult [5, 6].

A test’s diagnostic properties depends on the reference standard (the gold standard), the aim of the test, and the population. No gold standard exists. In this study, the reference standard was the Rome III criteria after exclusion of organic diseases. Most studies have used symptom-based criteria (e.g. Manning, Rome I, II and III) with a more or less complete exclusion of organic diseases [16, 28–31]. Other studies (like the one that validated the Rome III criteria) have used a not standardised definition [32]. The use of different reference standards makes comparisons of diagnostic tests for IBS nearly impossible.

Also, the aims of other studies have varied. Some studies aimed at separating patients with IBS-symptoms and organic diseases (like IBD) from patients with true IBS, some aimed at separating patients with IBS from healthy subjects, and some aimed at distinguishing subtypes of IBS [16, 28–31, 33]. The test results with one diagnostic marker vary depending on the aim of the study, as clearly shown by Ahmed et al. [16]. Most markers only differentiate IBS from organic diseases and not from healthy volunteers. At last, the participants in other studies have been selected from different populations, e.g. the general population, primary, secondary and tertiary care; healthy volunteers; different types of IBS and degree of symptoms; and patients with various organic disorders [16, 29, 31]. Comparisons of the results from studies of tests for the diagnosis IBS are unreliable without a strict control for the gold standard, the aims of the studies and the populations in the studies. Heterogeneity and design-related bias make comparisons of studies of diagnostic tests difficult [34, 35].

Ahmed et al. have studied the diagnostic accuracy of faecal volatile organic metabolites in IBS and showed that the results depended on the aim [16]. When the aim was to differentiate IBS from healthy individuals, as it was in the actual study, the sensitivity and specificity of the test were 90 and 80 % respectively. In the actual study, the marker was a simple difference between two SCFA, whereas Ahmed et al. used a complex discriminatory model [16]. Tests requiring resource consuming procedures (e.g. a large test battery, complex calculations, colonic transit time and faecal bile acid) have not been proven to be better than the more simple ones [16, 28–31].

### Strengths and limitations

The results of this small study were promising. If the measurement of SCFA in faeces turns out to be a valuable biomarker for IBS, it will be a simple test without invasive procedures and complicated calculations. The external validity of the actual study might be questioned since the subjects were recruited from a study comparing subjects with depression and unspecified neurological symptoms. However, the multivariable analyses did not indicate any confounding effects. Information about the diet, which has been shown to influence on the faecal microbiota, could have improved the study [17]. Neither was smoking habits recorded. The study evaluated only the diagnostic ability to differentiate IBS from healthy volunteers, and not IBS from organic diseases. Because the study had an exploratory design and a limited number of participants, the results need confirmation in a new, larger and hypotheses driven validation study.

## Conclusions

The study indicated that faecal SCFA could be a non-invasive, valid and reliable biomarker for the differentiation of IBS from healthy volunteers, particularly for the exclusion of the diagnosis.

### Availability of data and material

Case report forms (CRFs) on paper were used for collection of the clinical data, and all the CRFs are safely stored. The data were transferred manually to SPSS for statistical analyses. The data files are stored by Innlandet Hospital Trust, Brumunddal, Norway, on a server dedicated to research and with security according to the rules given by The Norwegian Data Protection Authority, P.O. Box 8177 Dep. NO-0034 Oslo, Norway. The data are available on request to the authors.
